# Paxillin’s nuclear switch: orchestrating gene splicing for brain plasticity

**DOI:** 10.1038/s44318-025-00596-w

**Published:** 2025-10-09

**Authors:** Souren Sadhukhan, Vijay K Tiwari

**Affiliations:** 1 Institute for Molecular Medicine, University of Southern Denmark, Odense M, Denmark; 2 Wellcome-Wolfson Institute for Experimental Medicine, School of Medicine, Dentistry, and Biomedical Science, Queens University Belfast, Belfast, UK; 3 Department of Clinical Genetics, Odense University Hospital, Odense C, Denmark; 4 Danish Institute for Advanced Study (DIAS), Odense M, Denmark

**Keywords:** Chromatin, Transcription & Genomics, Development, Neuroscience

## Abstract

Recent work in *The EMBO Journal* uncovers the regulatory role of nuclear paxillin in alternative splicing for synaptic refinement during brain development.

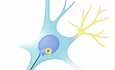

## Teaser

Paxillin, long known as a cytoplasmic scaffold protein involved in cell adhesion and migration, emerges in a new role as a nuclear regulator of alternative splicing in developing neurons. In this issue of *The EMBO Journal*, Chu and colleagues reveal that activity-dependent phosphorylation of paxillin at serine 119 promotes its nuclear translocation, enabling it to fine-tune gene splicing programs critical for synaptic refinement during sensitive periods of brain development.

**Figure 1:**
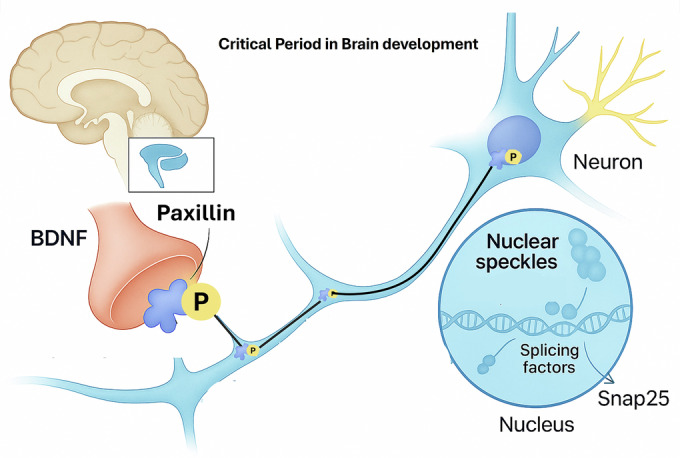
Paxillin’s activity-dependent nuclear role in neuronal splicing. This schematic illustrates the novel nuclear function of paxillin in neurons. Upon NMDA receptor activation or sensory stimulation, paxillin is phosphorylated at serine 119 (p-Paxillin^S119^), triggering its nuclear import via interaction with importin-β2. Once in the nucleus, p-Paxillin^S119^ localizes to nuclear speckles, where it interacts with RNA-binding proteins such as U2AFs, FUS, and NOVA1/2. This interaction modulates alternative splicing of synaptic genes, including Snap25, facilitating synaptic refinement and plasticity during sensitive periods of brain development. Disruption of this pathway leads to impaired synaptic transmission and memory deficits, highlighting paxillin’s critical role in bridging synaptic activity to genomic responses.

The brain’s ability to adapt and refine neural circuits during early postnatal life is fundamental to learning, memory, and overall cognitive function. This “sensitive period” of plasticity relies on precise molecular mechanisms that respond to sensory experiences, ensuring that neurons form appropriate connections. Alternative splicing (AS) plays a pivotal role here, generating diverse protein isoforms that modulate synaptic strength and morphology. However, the molecular switches that synchronize AS with neuronal activity have remained elusive. Chu et al. now position paxillin—a protein traditionally associated with focal adhesions—as a key activity-dependent mediator in the nucleus, bridging synaptic signals to genomic responses (Fig. 1).

In their study, the authors demonstrate that paxillin phosphorylated at serine 119 (p-Paxillin^S119^) is enriched in the developing mouse brain, peaking during postnatal weeks when sensory inputs refine circuits. This phosphorylation, triggered by NMDA receptor activation or sensory stimulation (e.g., whisker tactile input), facilitates paxillin’s nuclear import via proline-tyrosine nuclear localization signals and interaction with importin-β2 (Fig. 1). Once in the nucleus, p-Paxillin^S119^ localizes to nuclear speckles—hubs of splicing machinery—where it interacts with RNA-binding proteins like U2AFs, FUS, and NOVA1/2. This association modulates AS of synaptic genes, such as *Snap25*, shifting isoforms to support presynaptic function and plasticity.

These findings build on prior work showing paxillin’s nuclear presence in non-neuronal cells, where it influences transcription and proliferation (Dong et al, 2009; Ma and Hammes, 2018). In neurons, however, Chu et al uncover a novel, activity-gated mechanism: without S119 phosphorylation of paxillin, mice exhibit delayed *Snap25* isoform switching, impaired hippocampal synaptic transmission, and deficits in short-term memory. This echoes studies on activity-regulated proteomes, where synaptic inputs alter nuclear factors to drive transcription and splicing (Yap and Greenberg, 2018; Chen et al, 2021; Thakurela et al, 2015).

For instance, similar to how MeCP2 mutations disrupt splicing in neurodevelopmental disorders (Amir et al, 1999), paxillin’s role highlights how post-translational modifications can act as “molecular switches” for experience-dependent gene regulation.

This study expands paxillin’s repertoire beyond cytoskeletal dynamics, positioning it as a versatile regulator in genomic signaling. It aligns with emerging evidence of adhesion proteins moonlighting in the nucleus (Sathe et al, 2016) and underscores the importance of phosphoproteomics in uncovering hidden neuronal functions (Tsien, 2013). Broader implications extend to disorders like autism or schizophrenia, where splicing dysregulation during critical periods contributes to pathology (Quesnel-Vallières et al, 2016). Therapeutically, targeting paxillin phosphorylation could enhance plasticity in neurorehabilitation or mitigate age-related cognitive decline.

This work also raises intriguing questions: How does paxillin selectively influence certain splicing events? And could similar mechanisms operate in other cell types? By linking peripheral signals to nuclear alternative splicing, Chu et al provide a framework for understanding how experiences sculpt the brain, with potential ripple effects across developmental biology and beyond.
